# The prevalence of disorders causing disability in young adult males in Turkey between 2009-2011

**DOI:** 10.12669/pjms.295.3770

**Published:** 2013

**Authors:** Necmettin Kocak, Turker Turker, Ibrahim Aydin, Hakan Istanbulluoglu, Ramazan Akyildiz, Selim Kilic

**Affiliations:** 1 Dr. Necmettin Kocak, Turkish Coast Guard Command, Ankara, Turkey.; 2 Dr. Turker Turker, Assistant Professor, Department of Public Health, Gulhane Military Medical Academy, Ankara, Turkey.; 3 Dr. Ibrahim Aydin, Ministry of Defence, Ankara, Turkey.; 4 Dr. Hakan IstanbulluogluTurkish General Staff, Ankara, Turkey.; 5 Dr. Ramazan Akyildiz, Ministry of Defence, Ankara, Turkey.; 6 Dr. Selim Kilic, Professor, Department of Epidemiology, Gulhane Military Medical Academy, Ankara, Turkey.

**Keywords:** Disability, Hearing loss, Intellectual disability, Prevalence, Young adult men

## Abstract

***Objective:*** Disability is one of the significant problems that the public faces as regards social aspects, economics, public health and politics. Our aim was to review the prevalence of diseases causing disabilities in young adult men who are declared “unfit for military service” in Turkey after medical examination.

***Methods: ***We reviewed the prevalence of diseases among 113,175 young adult men who were referred for medical examination between 2009 and 2011.

***Results:*** Prevalence of unfitness for military service was 5.56% in 2009, 6.74% in 2010 and 6.77% in 2011. Leading causes for young adult men to be rejected from military service was intellectual disability 6.88, hearing loss 3.71, epilepsy 1.59, schizophrenia 1.54 and diabetes mellitus 1.47 per thousand people.

***Conclusion:*** Screening for the prevalence of disability conditions is an important data source for policies to be developed. Supporting such survey with community based studies in different populations in future shall be beneficial for improvement of policies in social and health fields.

## INTRODUCTION

Disability is one of the most important issues affecting the society in terms of social, economic, public health, and politics today.^1^ The International Classification of Functioning, Disability and Health defines disability as an umbrella term for impairments, activity limitations and participation restrictions. Disability is the interaction between individuals with a health condition and personal and environmental factors.^2^

It has been estimated that more than one billion people or 15% of the world population in, 2010 experienced some forms of disability in accordance with World Health Organization (WHO) criteria.^3^ Prevalence of disability has varied from country to country due to the differences in the criteria for assessing the disability all around the world. For instance, it has been found that 18.1% of men and 17.3% of women of all ages in Australia, 10.0% in the USA between 18 and 64 years, 3.8% in Ethiopia are classified as disabled or needs assistance for performing regular daily tasks.^4^^-^^6^

The number of handicapped people has increased worldwide. It has been estimated that 66.5% of the people with disability suffer from chronic diseases each year in low and middle-income countries.^7^ In some countries, trends such as environmental factors and traffic accidents, natural disasters, conflicts, diet and substance abuse have resulted in increased disability rates by influencing health conditions.^3^

It is compulsory for a man to perform their military service in Turkey.^8^ Every person is primarily examined in Recruiting Offices where two physicians are available in accordance with Health Ability Regulation (HAR) of Turkish Armed Forces (TAF) before they start their military service. During these health examinations, disabilities that will prevent these people from performing their military service are analyzed. Proceedings for cases such as loss of extremity, blindness etc. are made in Recruiting Offices, whereas cases requiring advanced examination such as intellectual disability, hearing loss, schizophrenia and epilepsy are performed in Military Hospitals. Health reports belonging to the men not suitable for Military service are collected in Health Department Directorate of National Defense Ministry (NDM). This data provides lot of useful information regarding diseases which cause impairments in the young adult male population of Turkey.

The objective of this study was to review the prevalence of diseases causing disabilities in young adult men from 2009 to 2011.

## METHODS

***Research Group: ***The number of people that applied for the first health examination for the compulsory military service between 2009 and 2011 was 1.777.500 from which 6.367% was rejected due to disabilities and disorders. We reviewed the health reports of 113.175 men who were declared “unfit for the military service” after examination for military service in Turkish Armed Forces (TAF) between 2009 and 2011. This cross-sectional study was conducted in August-September 2012. The number of people used in calculation of prevalence was 579.503 in 2009, 583.299 in 2010 and 614.698 in 2011.

***Administrative and Ethical Permissions: ***The data for this study was acquired from Surgeon General Office of Ministry of Defense. It was conducted after getting necessary permissions from Ethics Committee of Gulhane Military Medical Faculty (GMMF).

***Descriptions in the Survey: ***Decision regarding health conditions in TAF are made in accordance with the criteria defined in HAR. Diseases and disorders in HAR are classified as functional and described in the titles between 1 and 70. 

“Training Hospitals” are tertiary military hospitals such as GMMF Ankara and Haydarpasa Istanbul, whereas “regional hospitals” are military hospitals with 600 beds, and other military hospitals are classified as “secondary stage military hospitals.” Recruiting Office consists of at least two doctors and the administrative staff. These offices transfer the men to military hospitals if a patient has a disease observed through external physical examination or is too weak to go to the hospital.

***Evaluating the data and statistical analysis: ***The cities recruiting offices are grouped in accordance with the quinary zoning system prepared by Turkish Statistical Institute.^9^ Cities are classified as West, South, Middle, North and East in accordance with this zoning system. Participants’ ages are classified as “19, 20, 21-24 and over 25” as the people are taken into health examination between 19 and 20 ages.

The data collected is entered into computers via SPSS 15.0 statistics program and then analyzed. Frequency and percentage are used for discrete data as descriptive statistics and mean±standard deviation are used for continuous variables.

## RESULTS

In this study, health reports belonging to 113.175 young adult men, who were declared “unfit for military service” between 2009 and 2011, were reviewed. It showed prevalence of unfitness for military service was 5.56% in 2009, 6.74% in 2010 and 6.77% in 2011. 

We also found out that the most common age group was the 19 year-old with a health deficiency proportion of (28.7%), the most common region in means of recruiting office was the Eastern Anatolia Region at (28.9%) and the hospital group at which maximum examinations are conducted is secondary military hospitals at (45.7%) (Table-I).

In this study the subject’s ineligibility for military service was divided into three classifications: disability, non-disability and unclassified conditions.

**Fig.1:**
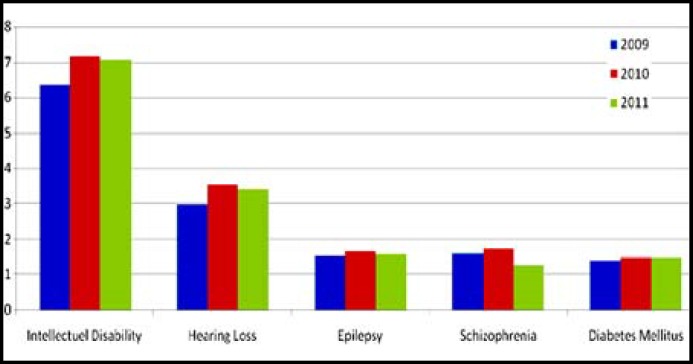
The change in prevalence that causes the circumstances of disability between 2009-2011 (per thousand) (Ankara 2012).

The most common conditions that cause disability in young adult males in Turkey was intellectual disability, hearing loss, epilepsy, schizophrenia and diabetes mellitus with a prevalence of 6.88, 3.71, 1.59, 1.54 and 1.47 per thousand people respectively. The top three non-disability conditions observed in this study were obesity, refraction disorders and lack of weight (Table-II). The change in prevalence that causes the circumstances of disability between 2009-2011 is given in Fig.1.

**Table-I:** Distribution of cases by age group, region and hospital (Ankara, 2012).

		*2009*		*2010*		*2011*		*Total*	
		*n*	*%*	*n*	*%*	*n*	*%*	*n*	*%*
Age groups	19	10012	31.1	10896	27.7	11546	27.7	32454	28.7
	20	5177	16.1	7882	20.0	8329	20.0	21388	18.9
	21-24	8505	26.4	10104	25.8	11215	26.9	29824	26.4
	25 and above	8508	26.4	10451	26.5	10550	25.4	29509	26.0
Region	West	6372	19,8	7949	20,2	7960	19,1	22281	19,7
	South	3501	10,9	4369	11,1	4450	10,7	12320	10,9
	Central	8703	27,0	10465	26,6	11246	27,0	30414	26,9
	North	4418	13,7	5374	13,7	5651	13,6	15443	13,6
	East	9208	28,6	11176	28,4	12333	29,6	32717	28,9
Hospital	Training H.	4734	14,7	5891	15,0	6736	16,2	17361	15,3
	Regional H.	8727	27,1	11146	28,3	12870	30,9	32743	28,9
	Secondary stage H.	15245	47,3	18405	46,8	18121	43,5	51771	45,7
	Military council	3103	9,6	3216	8,2	3456	8,3	9775	8,6
	Other*	393	1,2	675	1,7	457	1,1	1525	1,3
Total		32202	100,0	39333	100,0	41640	100,0	113175	100,0

*. Decisions from civil or foreign hospitals

**Table-II:** Prevalence of the first ten diagnoses (Ankara 2012

*Diagnoses*	*2009*			*2010*			*2011*			*Total*		
	*n*	*%*	*%*_0_ *	*n*	*%*	*%*_0_ *	*n*	*%*	*%*_0_ *	*n*	*%*	*%*_0_ *
***Disability conditions***												
Intellectuel disability	3692	11.5	6.37	4182	10.6	7.17	4353	10.5	7.08	12227	10.8	6.88
Hearing loss	1922	6.0	3.32	2305	5.8	3.95	2373	5.72	3.86	6600	5.83	3.71
Epilepsy	887	2.8	1.53	975	2.5	1.67	962	2.3	1.57	2824	2.5	1.59
Schizophrenia	923	2.9	1.59	1017	2.6	1.74	788	1.9	1.28	2728	2.4	1.54
Diabetes mellitus	817	2.5	1.41	875	2.2	1.50	916	2.2	1.49	2608	2.3	1.47
***Non-disability conditions***												
Obesity	4710	14.7	8.13	7678	19.5	13.16	8447	20.3	13.74	20835	18.4	11.72
Refraction disorders	1664	5.2	2.87	2005	5.1	3.44	2292	5.5	3.73	5961	5.3	3.35
Lack of weight	684	2.1	1.18	1222	3.1	2.10	1351	3.3	2.20	3257	2.9	1.73
***Unclassified conditions***												
Central nervous system diseases	1647	5.1	2.84	1909	4.9	3.27	2002	4.8	3.26	5558	4.9	3.13
Peripheral nervous system diseases	720	2.2	1.24	683	1.7	1.17	727	1.8	1.18	2130	1.9	1.20

*prevalence

## DISCUSSION

In this study the prevalence of unfitness for military service was found to be 5.56% in 2009, 6.74% in 2010 and 6.77% in 2011. In the study by Kocoglu in Turkey, it has been reported that the unfitness for military service rate was 4.25%, 5.35% and 4.46 % in people born in 1945, 1950 and 1955, respectively.^10^ Study by Kilic, on 21.985 young adult males showed the rate of unfitness for military service was 4.57%.^11^ In our study the prevalence of incapable young adult males is found to be higher than other studies conducted in Turkey. These results suggest that rates of unfitness for military service have increased in akin with the increase in the prevalence of obesity in public. In a survey conducted by Taanila et al on 1411 young adult males who were about to be accepted into their military service in Finland, it was observed that 9.4% of the participants were rejected due to their impairments.^12^

Prevalence of intellectual disability is 1% worldwide and it is observed in men more frequently than in women.^13^ In our survey, intellectual disability was found to be 6.37 per thousand in 2009, 7.17 per thousand in 2010 and 7.08 per thousand in 2011. Furthermore, the prevalence in other surveys conducted in Saudi Arabia^14^ and the USA^15^ is similar to ours. However, in survey conducted in Pakistan^16^ higher rates of impairments have been detected.

The second most common cause of disability in the world has been reported as hearing loss.^17^ The same results were obtained in our study. In this study, hearing loss after (40dB or more) has become an impairment and sequelae over the years; the pervasiveness of hearing loss per thousand for the year 2009 was 3.32, 3.95 per thousand for the year 2010 and 3.86 per thousand for the year 2011 have been observed. Kilic’s study showed a moderate and severe hearing loss of 1.7 per thousand and severe hearing loss and deafness was found to be 0.56 per thousand.^11^ According to the American Community Survey, in 2010, for men aged between 16 and 20 in the United States, deafness and hearing loss was at 0.7%, and 2.8% for men aged between 21 and 64.^5^

Epilepsy is a chronic non-communicable disorder of the brain that affects people of all ages. The estimated proportion of the general population with active epilepsy is between 4 to 10 per 1000 people. However, some studies in developing countries suggest that the proportion is between 6 to 10 per 1000. Around 50 million people worldwide have epilepsy.^18^ In our survey, the prevalence of epilepsy was found at 1.53 per thousand in 2009, 1.67 per thousand in 2010 and 1.57 per thousand in 2011. Ebrahimi et al carried out a survey in Iran and observed an epilepsy rate of 7.87 per thousand.^19^ An analysis by the Centers for Disease Control and Prevention (CDC) in 2010, suggested a epilepsy rate of ten per thousand in the United States.^20^

Schizophrenia is a severe form of mental illness affecting about 7 per thousand of the adult population, mostly in the age group of 15-35 years. Although the incidence is low (3 per 10,000), the prevalence is high due to chronicity.^21^ In our survey, prevalence of schizophrenia was found to be 1.59 per thousand in 2009, 1.74 per thousand in 2010 and 1.28 per thousand in 2011. In Italy, Tansella et al reported its prevalence to be 1.3 for males and 1.1 for females per thousand.^22^ In England, Freeman and Alpert found rates per thousand to be 7.0 in males and 6.6 in females.^23^

Developments in technology, sedentary lifestyle and obesity diabetes mellitus has increased worldwide. In the year 2000 there was an estimated of 151 million people with diabetes in the world, which is likely to double by the year 2025 reaching about 300 million.^24^^,^^25^

In our study, prevalence of diabetes mellitus was found to be 1.41 per thousand in 2009, 1.50 per thousand in 2010 and 1.49 per thousand in 2011. In Kilic’s study, the diabetes mellitus prevalence was 0.5 per thousand people.^11^ This increase in obesity in society, especially in the recent years is thought to be responsible for the increased prevalence of diabetes.

There are a few factors that have led to the observation of lower rates of schizophrenia, epilepsy and diabetes mellitus in our study. Most importantly, 64% of the age group being observed was between the ages of 19-20, furthermore, military service is considered to be an honour by patients and their families and diseases are commonly kept a secret. Evaluating numerous people for all diseases in a very short time period is also considered to be beneficial leading to proper diagnosis of diseases.

The diagnoses of the cases are transferred to the computer by using the codes in HAR which was a restriction of this survey. Diseases in HAR have been classified functionally. Due to functional classification especially in some disease groups, disease rates resulting in disability could not be calculated.

Our survey has been conducted by reviewing health reports of 113,175 people who have been rejected from military service. The large survey population of this study can be deemed a strength of this study. We also observed prevalence of disability conditions among young adult men and its change between 2009 and 2011. 

## CONCLUSION

This study showed that, prevalence of unfitness for military service in Turkey was 5.56% in 2009, 6.74% in 2010 and 6.77% in 2011. It shows a slight gradual increase every year. It also showed that the levels of hearing loss in Turkey was similar to other countries, intellectual disability in Turkey similar to the prevalence in other countries, and Turkey has lower rates of schizophrenia, epilepsy and diabetes mellitus. Detecting the prevalence of disability conditions is a significant data source which can be used by policy makers to improve health & social services besides conducting further community based studies.
